# Severe hypercalcemia caused by parathyroid hormone in a rectal cancer metastasis: a case report

**DOI:** 10.1186/s12902-020-00664-8

**Published:** 2021-01-07

**Authors:** Vegard Heimly Brun, Erik Knutsen, Helge Stenvold, Hanne Halvorsen

**Affiliations:** 1 Department of Breast- and Endocrine Surgery, University Hospital of North Norway, Sykehusvegen 38, 9019 Tromsø, Norway; 2 UiT The Arctic University of Norway, Faculty of Health Sciences, Hansine Hansens veg 18, 9019 Tromsø, Norway; 3 Department of Oncology, University Hospital of North Norway, Sykehusvegen 38, 9019 Tromsø, Norway; 4 Department of Clinical Pathology, University Hospital of North Norway, Sykehusvegen 38, 9019 Tromsø, Norway

**Keywords:** Parathyroid hormone, Hypercalcemia, Colorectal cancer, Metastasis, Case report

## Abstract

**Background:**

Hypercalcemia of malignancy is relatively common in several cancers. However, in colorectal cancer, paraneoplastic phenomena that cause hypercalcemia is uncommon. In the few cases that are reported, secretion of parathyroid hormone-related peptide mediates the effect. We describe the first case of severe hypercalcemia mediated by intact parathyroid hormone secretion from a bone metastasis of colorectal origin. This was a diagnostic and therapeutic challenge.

**Case presentation:**

A 68-year-old male treated for rectal adenocarcinoma 10 years earlier developed a bone metastasis. After initial treatment of the metastasis with surgery and irradiation, he developed a relapse with severe hypercalcemia and corresponding elevated parathyroid hormone levels. The workup showed no signs of parathyroid adenomas, but the metastasis produced intact parathyroid hormone. The hypercalcemia was successfully treated by irradiation and osteoclast inhibitor, and the patient received chemotherapy. Survival was 24 months from the onset of hypercalcemia.

**Conclusions:**

Proper diagnosis of the uncommon endocrine disturbance allowed targeted therapy and avoidance of neck exploration for wrongly suspecting primary hyperparathyroidism. Intact parathyroid hormone should be measured in cases of malignant hypercalcemia.

**Supplementary Information:**

The online version contains supplementary material available at 10.1186/s12902-020-00664-8.

## Background

Hypercalcemia of malignancy occurs in approximately 20–30% of all cancers [1], and is associated with poor prognosis. Thirty-day-mortality in cancer-related hypercalcemia can be as high as 50% [2]. Medical treatment may require hospitalization and have severe side effects [3]. Although the underlying cause can be osteolytic metastases, hypercalcemia of malignancy is usually caused by secretion of an active peptide such as parathyroid hormone-related peptide (PTHrP), parathyroid hormone (PTH) or calcitriol [4, 5]. Secretion of PTHrP or ectopic PTH has been described in neuroendocrine tumors and a range of carcinomas, leukemia, lymphoma and rhabdomyosarcoma. The carcinomas known to cause these endocrine paraneoplastic effects are squamous cell, renal, bladder, breast, ovary, lung, thyroid, neuroectodermal and pancreatic carcinomas. Hypercalcemia in colorectal adenocarcinomas is rare, and it is usually caused by secretion of PTHrP. To our knowledge, this is the first report of intact PTH production from a colorectal bone metastasis.

## Case presentation

A 68 years old male had undergone total mesorectal excision 10 years before a skeletal metastasis of the lower leg was diagnosed. The patient was otherwise healthy except mild hypertension and there was no familial accumulation of endocrine or malignant diseases. Histology from the primary surgery showed a 5 cm intermediately differentiated adenocarcinoma of the rectum with no signs of neuroendocrine biology. The tumor was CK7 negative and CK20 positive. Distance to the nearest circumferential margin was 3 mm, and end resection margins were clear. There was no infiltration in the removed lymph nodes nor signs of distant metastases, thus the final TNM status was pT3N0M0. Total calcium was normal (2.37 mmol/L). Carcinoembryonic antigen (CEA) dropped from 18.0 μg/L preoperatively to 1.5 μg/L after surgery. No neo-adjuvant or adjuvant radiochemotherapy was given during the primary treatment. He underwent standard follow-up regimen with bloodwork, rectoscopies, ultrasound of the liver and chest x-ray for 4 years without any signs of relapse. CEA values were stable under 2.0 μg/L and only 1.0 μg/L when follow-up was completed.

**Fig. 1:**
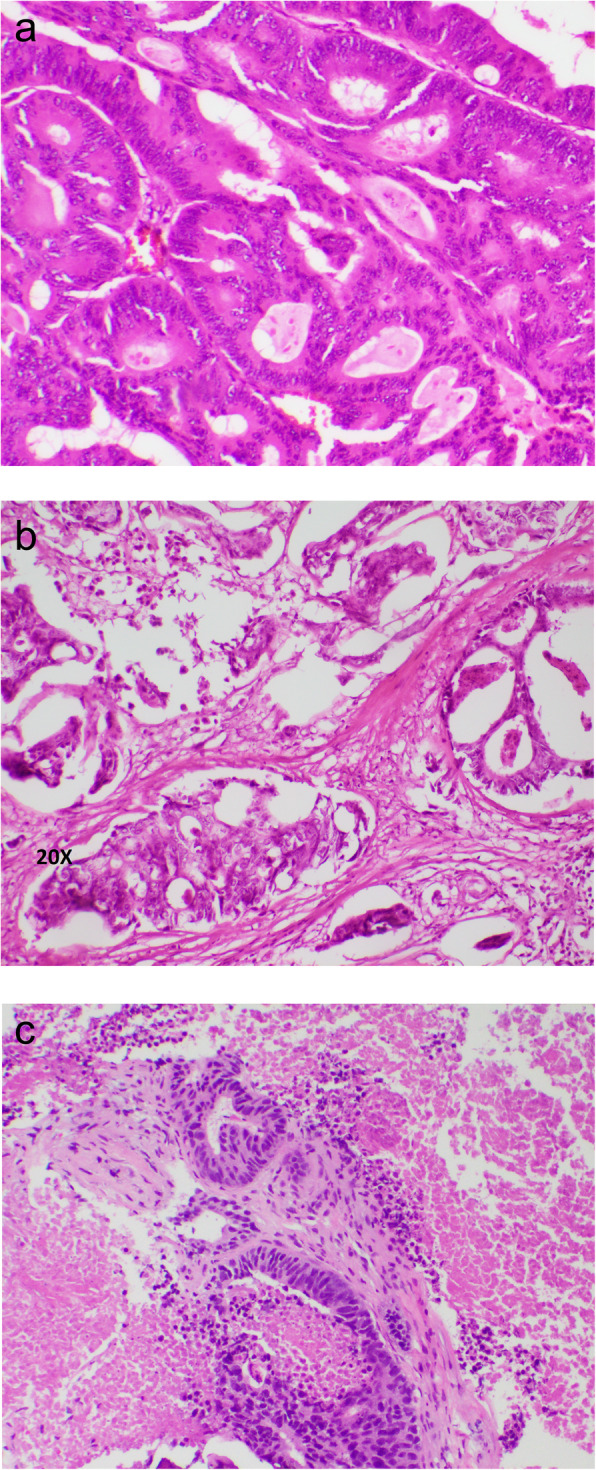
Hematoxylin and eosin staining showed architecture typical for adenocarcinoma. Representative sample form the rectal resection (**a**), resection of fibula head (**b**), and from the fibula biopsy taken after onset of hypercalcemia (**c**)

Ten years after surgery, the patient presented with a tumor in the lower leg. X-ray and magnetic resonance imaging showed a 4.4 cm osteolytic lesion of the fibular head, in addition to a 4.7 cm tumor in the left lung and paratracheal pathological lymph nodes. Biopsies of the fibular head confirmed adenocarcinoma negative for CK7 and positive for CK20, and the pathologist concluded metastasis from the rectal cancer. The histological pattern was similar to the previously resected rectal tumor (Fig. 1). CEA had now increased to 15.4 μg/L. He was treated by radiation therapy to the leg (8 Gy × 1) and commenced chemotherapy (fluorouracil, irinotecan, calcium folinate and bevacizumab). His lung pathology stabilized during this treatment and he reported less pain from the leg after the radiation therapy. However, the fibular tumor progressed radiologically and was 7 cm in diameter when it was resected 15 months after discovery. Resection margins were uncertain, and he developed a wound infection with a chronic fistula to the skin.

**Fig. 2:**
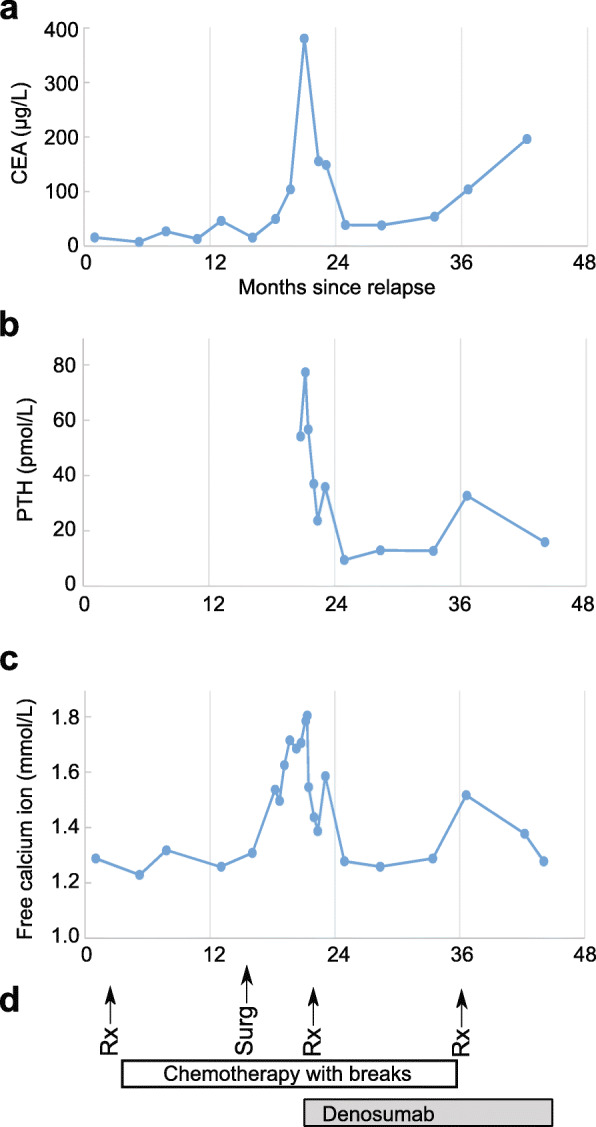
Levels of carcinoembryonic antigen (CEA, **a**), parathyroid hormone (PTH, **b**) and free calcium (**c**) after diagnosing rectal cancer relapse. **d** Outline of main treatment events. Rx: radiotherapy towards the fibula. Surg: Surgical resection of the fibular head

Three months later, the patient presented with hypercalcemia after being normocalcemic during all previous follow-up. Due to rapidly growing inguinal and retroperitoneal lymph nodes, he started treatment with fluorouracil, leucovorin, and oxaliplatin (FLOX). CT-scan showed stable disease after three cycles of therapy. Bloodwork revealed that CEA had increased markedly to 380 μg/L. He developed severe hypercalcemia with free ionized calcium reaching 1.81 mmol/L (total calcium 3.33 mmol/L), concurrent with peaks in CEA and PTH levels (Fig. 2). Creatinine and estimated GFR was normal, phosphorus was low (0.62 mmol/L), and 1,25-dihydroxyvitamin D was low (24 nmol/L). He had no arrhythmias or other clinical symptoms related to the hypercalcemia. Fluid therapy and zoledronic acid had only short-lasting effect on the calcium levels. PTHrP was negative, but intact PTH was elevated to 54.4 pmol/L (normal range 1.1–7.5 pmol/L). The lab used a standard two-site immunoassay for measurement of the 84 amino acid long PTH, requiring binding in both the N and C terminals for detection. The high PTH led us to consider primary hyperparathyroidism as a differential diagnosis. Ultrasound of the neck and parathyroid scintigraphy with 900 MBq of 99mTc-methoxyisobutylisonitrile (MIBI) showed no signs of parathyroid adenomas. The diagnostic challenge was to distinguish primary hyperparathyroidism from ectopic PTH production in a colorectal bone metastasis or potentially another tumor.

**Fig. 3:**
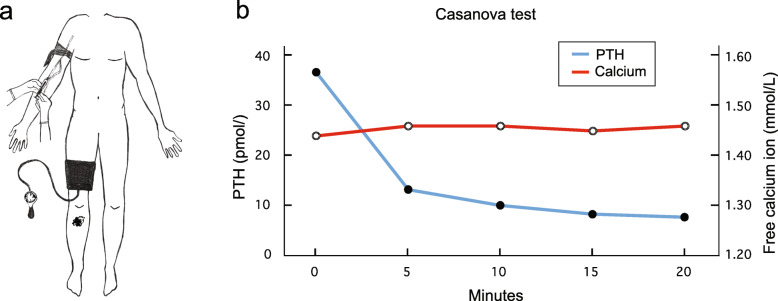
Modified Casanova test to determine the source of PTH secretion. **a** A blood pressure cuff excluded the bone metastasis in the right fibula from circulation while systemic PTH and calcium levels were measured every 5 min. **b** A marked drop in PTH (blue line) during the 20 min ischemia confirmed PTH production in the right lower leg

To investigate if the tumor of the fibular head was responsible for the PTH elevation, we biopsied the tumor and stained for PTH. No significant staining was observed, using monoclonal antibody immunohistochemistry. Because such a large tumor can contain different clones of cells, a negative biopsy does not rule out that PTH could be produced from a part of the tumor not represented in the biopsy. Also, PTH immunohistochemistry on bone biopsies could be false negative for unknown technical reasons. We therefore measured blood PTH levels while excluding the lower right leg from the circulation by use of a blood pressure cuff on the thigh (modified Casanova test [6], Fig. 3a). PTH levels measured in blood samples from the arm fell rapidly during the 20 min of right leg ischemia, consistent with local PTH production from the fibular tumor (Fig. 3b).

We concluded that the diagnosis was hypercalcemia caused by PTH secretion from the bone metastasis in the fibular head, and focused further treatment against the fibular tumor. The osteoclast inhibitor denosumab 120 mg was administered subcutaneously and further radiotherapy was given to the lower leg (3 Gy × 10). This made free calcium level stabilize at values around 1.40 mmol/L, and PTH was also attenuated (Fig. 2). The patient received further cycles with FLIRI/bevacizumab until a year before his death. Six months before the patient died, another surge in PTH and calcium levels arose. Now, the patient received another course of radiotherapy (4 Gy × 5) towards his fibula, followed by a drop in PTH levels and normalization of calcium levels. No adverse events or side effects were reported by the patients or treating clinicians. The patient did not develop further episodes of severe hypercalcemia during the remainder of his life and died approximately 24 months after the first incident of severe hypercalcemia, due to disseminated disease involving skin, lymph nodes, lung, pancreas and brain.

**Fig. 4:**
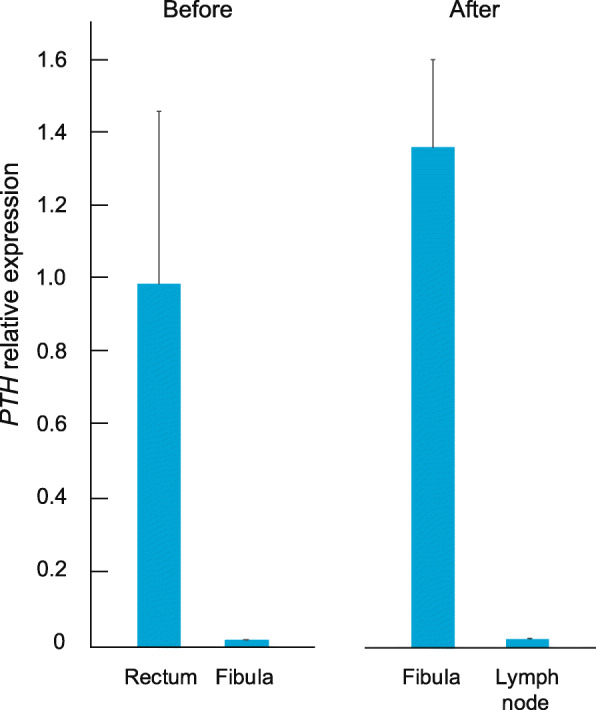
Relative *PTH* mRNA expression in biopsies *before* and *after* onset of hypercalcemia. Mean values + standard deviations of three replicates are presented as fold change relative to rectum tumor. The two leftmost bars show detectable *PTH* expression levels in the resected rectum tumor, but not in the first fibula biopsy, taken 10 years later, before onset of hypercalcemia. The two rightmost bars represent expression levels 1 year later, after onset of hypercalcemia, in the relapsed fibular tumor and in an ipsilateral inguinal lymph node

As immunohistochemistry failed to show PTH in the fibular bone metastasis, we analyzed messenger ribonucleic acid (mRNA) from the same biopsies post mortem. Despite the long interval from biopsy to RNA extraction (5–16 years), we were able to extract total RNA from formalin-fixed paraffine embedded (FFPE) tissue sections and quantified PTH by reverse transcriptase quantitative polymerase chain reaction (RT-qPCR)^1^. We found evidence of *PTH* transcription in both the original rectal specimen and in the fibular biopsy taken *after* onset of hypercalcemia, but not in the fibular biopsy that was taken *before* onset of hypercalcemia, nor in ipsilateral inguinal lymph node metastasis (Fig. 4). This is consistent with development of clinically significant PTH production within the fibular metastasis, after local recurrence and radiation therapy to the lower leg.

## Discussion and conclusion

The case report is, to our knowledge, the first to describe production of intact PTH in a bone metastasis from rectal adenocarcinoma. The case illustrates the diagnostic and therapeutic challenges, and describes a multimodal treatment during the relatively long survival of 24 months from the diagnosis of severe hypercalcemia. A review of malignant hypercalcemia in colorectal cancer described 29 cases with elevated PTHrP secretion [7], but none had hypersecretion of intact PTH. On the contrary, PTH levels are usually suppressed as a consequence of the hypercalcemia. PTHrP secretion is more common in tumors with neuroendocrine differentiation [8], but we did not find signs of neuroendocrine differentiation in the specimens examined from our patient.

In our case, the evidence that PTH secretion was indeed from the metastasis and not from a parathyroid adenoma or hyperplasia, are the modified Casanova test and post mortem measurements of *PTH* mRNA levels in biopsies. The systemic PTH level dropped when the blood circulation to the lower leg was excluded for 20 min. We could not demonstrate PTH production in the tumor by immunohistochemistry, however, the biopsy from the fibular metastasis had high expression of *PTH* mRNA. We did not find evidence of other alternative PTH producing tumors in the lower leg on computer tomography scans, FDG-PET/CT scan or ultrasound scans that were made shortly after onset of hypercalcemia.

It is tempting to speculate that the irradiation therapy that was administered shortly after the relapse induced a transformation of the tumor cells to produce PTH, although there was a > 1 year interval between radiation and hypercalcemia. Such transformation is not reported previously for adenocarcinomas, but the potential for radiation to induce malignant transformations is well-known [9, 10]. More likely, the treatment could have led to selection of sub-clones with neuroendocrine properties, as the original rectal tumor showed evidence of *PTH* transcription. We did not see obvious transformation or dedifferentiation of the tumor cells upon examination and re-examination of all histological specimens. The pathological reports were consistent with primary rectal adenocarcinoma with similar morphology in the fibula specimen and biopsies.

To conclude, hypercalcemia of malignancy is relatively common in general, but rare in colorectal cancer. PTH should be measured in addition to PTHrP and calcitriol in these cases to allow tailored treatment. The differential diagnosis of primary hyperparathyroidism should be considered.

## Supplementary Information

**Additional file 1.**

## Data Availability

Not applicable.

## Abbreviations

PTH: Parathyroid hormone
PTHrP: Parathyroid hormone-related peptide
FLOX: Fluorouracil, leucovorin, oxaliplatin
FLIRI: Fluorouracil, folinic acid, irinotecan
CEA: Carcinoembryonic antigen
TNM: Tumor, nodes, metastasis
FDG-PET: Fluorodeoxyglucose positron emission tomography
FFPE: Formalin-fixed paraffin embedded
mRNA: Messenger ribonucleic acid
cDNA: Complementary deoxyribonucleic acid
RT-qPCR: Real-time quantitative polymerase chain reaction

## Footnotes

1 *RNA Isolation, cDNA Synthesis, and RT-qPCR. * Total RNA was isolated from FFPE tissue sections using the High Pure FFPE tissue RNA Isolation kit (Roche) according to the manufacturer’s recommendation. RNA concentration was measured by Qubit (Thermo Fisher Scientific). Complementary deoxyribonucleic acid (cDNA) synthesis of total RNA was performed with SuperScript™ IV Reverse Transcriptase (ThermoFisher Scientific). 2.5 μM of random hexamer primer (ThermoFisher Scientific) and approximately 80 ng of template were used for the reaction. Total RNA was denaturated at 65 °C for 5 min, and cDNA was synthesized at 53 °C for 10 min. For real-time quantitative polymerase chain reaction (RT-qPCR), cDNA was mixed with FastStart Essential DNA Green Master (Roche Life Science) and 0.25 μM forward and reverse primer. All primer sequences are provided in Supplementary Table 1. The LightCycler® 96 was used for quantification, and the ΔΔCq-method was used to calculate fold change using the geometric mean of housekeeping genes (*GAPDH* and *RPLPO)* as internal reference.
