# Supplementary material for: Severe hypercalcemia caused by parathyroid hormone in a rectal cancer metastasis: a case report
Source: BMC Endocr Disord. 2021 Jan 7;21:4. doi: 10.1186/s12902-020-00664-8 (PMC7792014; doi:10.1186/s12902-020-00664-8)
Supplement: Supplementary file 1 — Additional file 1. [file 12902_2020_664_MOESM1_ESM.docx]

Supplementary Table 1:

| **Gene** | **Forward primer** | **Reverse primer** |
| --- | --- | --- |
| *GAPDH* | GAGCGAGATCCCTCCAAAAT | AAATGAGCCCCAGCCTTCT |
| *RPLPO* | GCTGCTGCCCGTGCTGGTG | TGGTGCCCCTGGAGATTTTAGTGG |
| *PTH* | GGCAGACAAAGCTGATGTGAAT | AGCAGCATGTATTGTTGCCCT |
